# Triple negative breast cancer in Moroccan women: clinicopathological and therapeutic study at the National Institute of Oncology

**DOI:** 10.1186/1472-6874-12-35

**Published:** 2012-10-07

**Authors:** Ghizlane Rais, Soundouss Raissouni, Meryem Aitelhaj, Fadoi Rais, Sara Naciri, Siham Khoyaali, Halima Abahssain, Youssef Bensouda, Basma Khannoussi, Hind Mrabti, Hassan Errihani

**Affiliations:** 1 Medical oncology Department, National Institute of Oncology, Rabat, Morocco; 2 Department of radiotherapy, National Institute of Oncology, Rabat, Morocco; 3 Pathology department, National Institute of Oncology, Rabat, Morocco

## Abstract

**Background:**

Triple-negative breast cancer (TNBC) is defined by the lack of estrogen receptor (ER), progesterone receptor (PR), and human epidermal growth factor receptor 2 (HER-2) expression. This is an aggressive malignancy with a poor prognosis despite the high rates of response to chemotherapy. The aim of this study is to determine the clinicopathological, therapeutic features and outcomes associated with this type of breast cancer.

**Methods:**

This is a retrospective study of confirmed triple negative breast cancer females collected at the National institute of oncology of Rabat in Morocco, between January 2007 and December 2008. Epidemiological, clinical, histological, therapeutic and evolutive data were analyzed. OS and DFS rates were estimated by Kaplan-Meier analysis.

**Results:**

A total of one 152 patients with breast cancer, were identified as having triple-negative breast cancer (16,5%). The median age at diagnosis was 46 years. 130 patients (86%) had infiltrating ductal carcinoma and thirteen had medullar carcinoma (9%). 84 cases (55%) were grade III Scarff-Bloom-Richardson (SBR). 48 % had positive lymph nodes, and 5 % had distant metastases at diagnosis. According TNM staging, 12 patients (8%) had stage I, 90 patients (60%) had stage II and the 43(28%) had stage III. 145 patients received surgery. 41 (28%) had conservative surgery and 104 (72%) received radical mastectomy with axillary lymph nodes dissection. 14 patients with advanced tumors or inflammatory breast cancer have received neoadjuvant chemotherapy and four patients (28%) had complete pathologic response. From 131 patients how received adjuvant chemotherapy, 99 patients (75,5%) had Anthracycline based chemotherapy) and 27 patients (20,6%) had sequential Anthracycline and docetaxel,. Seven patients with metastatic disease received anthracycline-based regimen in the first line metastatic chemotherapy. The median follow-up time was 46 months (range 6,1 -60 months). Overall survival at 5 years for all patients was 76,5%.

**Conclusion:**

These results suggest that most TNBC characteristics in Moroccan patients are in accordance with literature data, especially concerning young age at diagnosis high grade tumors, advanced stage at diagnosis, and short time to relapse. Although the high response rate to chemotherapy, the overall prognosis of this subset of tumors remains poor.

## Background

Breast cancer affected an estimated 232,620 women and men in 2011, and was responsible for 39,970 deaths during the same year, in the United States [1]. It is now widely recognized that is a heterogeneous disease composed of different subtypes, characterized by their different clinic-pathological features, prognoses and responses to treatment [2,3]. Recently, five distinct gene expression profile-based “intrinsic” subtypes were identified by c DNA microarray analysis. Luminal A (ER+and/or progesterone receptor positive [PR+], HER2−,low Ki67), luminal B (ER+ and/or PR+, HER2+ (or HER2- with high Ki67)), basal-like (ER−, PR−, HER2−, cytokeratin 5/6 positive, and/or HER1+), HER2+/ER− (ER−, PR−, an HER2+), and unclassified (negative for all 5 markers) [2-4]. [4]. Triple-negative breast cancer (TNBC) is defined by the lack of estrogen receptor (ER), progesterone receptor (PR), and human epidermal growth factor receptor 2 (HER-2) expression [4]. It is important to clarify that the terms “triple negative” and “basal-like” are not completely synonymous, illustrating an approximately 20%–30% discordance across several studies [5]. The term triple negative refers to the immunohistochemical classification, whereas the basal-like subtype is defined via gene expression microarray analysis. In TNBC ,we can distinguish between two groups: basal-like (ER-, PR-,Her2-, cytokeratin (CK) 5/6+ and/or Her1+) and unclassified subtype (ER-, PR-, Her2-, Her1- and CK5/6-). The incomplete overlap between basal and TN breast cancers could translate true differences in their biology. Triple-negative tumors represent a more heterogeneous group than basal tumors, and include basal and non-basal tumors very different both at the histoclinical and molecular level [5].

Approximately 15-20% of breast cancers are triple negative [6,7], the majority of them are from the basal-like subtype. TNBC occurs disproportionately in younger women (<50 years) [6-10], in African-American women [11,12], and in carriers of BRCA1 [6]. To date, studies on Moroccan patients with TNBC have been limited by small sample sizes and short follow-up times [13]. To some extent, this is because the TNBC is based on immunohistochemical staining of tumor slides, to identify the overexpression of HER 2 neu (HER2) and these were not in general clinical use before 2007. The specific aim of this review was to characterize this population in clinical terms. However, we tried to determine retrospectively the incidence and survival of TNBC patients in the National Institute of Oncology in Morocco.

## Methods

The National Institute of Oncology database was used to identify patients with triple negative breast cancer between January 2007 and December 2008. We excluded from the study patients who had not follow up after initial diagnosis. The scientific comity of National Institute of Oncology approved the retrospective review of the medical records for the purposes of the current study. Breast carcinoma diagnosis was made by biopsy of the breast tumor. Patient information was recorded at accrual and included details about the patient’s age at diagnosis (in years), tumor grade, lymph node status, pathologic tumor size in centimeters, and treatment (surgery, chemotherapy, and radiation treatment). Tumor staging was carried out according to the TNM classification 2002 modified in 2003. Histological tumor grading was performed using the Scarff Bloom and Richardson (SBR) histological system. Immunohistochemistry: Immunohistochemical analysis to determine estrogen (ER) and progesterone receptor (PR) status was performed using standard procedures on 4-μm sections of paraffin embedded tissue specimens stained with the monoclonal antibodies 6F11 and 1A6 for ER and PR, respectively. Nuclear staining 10% was considered a positive result. The Hercept test was realized in the institute since 2007, subsequently, HER2 status was not routinely determined for all patient treatment during the course of this study. Assays are scored with a 4-tiered system (0–3+). HER2 positivity was defined as strong complete membrane staining in at least 10% of tumor cells. Patients were considered HER2-positive if they had immunohistochemistry (IHC) 3+ by DAKO herceptest. Tumors exhibiting equivocal HER-2 expression, denoted as 2+ membranous staining of tumor cells, are confirmed by fluorescence in situ hybridization (FISH) at an outside laboratory. A signal ratios (HER2: CEP17) of ≥ 2.2 were classified as amplified. In the absence of positive FISH data, tumors scored 2+ by IHC were considered negative for HER-2. Nine hundred eighteen patients of 2004 patients (45,8%) had sufficient details on hormone receptors and HER2 for classification.

### Treatment

Patients with local disease had received corresponding local treatments (surgery plus radiotherapy) and systemic treatments (mainly adjuvant and/or neoadjuvant chemotherapy). The main surgical operations included radical mastectomy (Patey type mastectomy) and breast conserving surgery when permitted by tumor size according to the judgment of the multidisciplinary care team. Patients how had metastatic disease at diagnosis received mono or poly chemotherapy based on the characteristics of the tumors and the aggressiveness of the disease. These patients have received palliative radiotherapy if indicated.

In our institute and in the study period, adjuvant chemotherapy in TNBC patients was indicated in the case of tumor size greater than or equal to 2 cm, positive nodal status, grade 2 or 3 SBR, and age ≤ 35 years. Neo-adjuvant chemotherapy was giving in patients with inflammatory or locally advanced breast cancer. For the patients who did not receive adjuvant chemotherapy, they have received neo adjuvant chemotherapy or they did not return after surgery for personal reasons.

For patients receiving neoadjuvant chemotherapy, Chevalier classification was used to classify histological response to neo-adjuvant chemotherapy in the breast [14]. A pathologic complete response (pCR) was defined as no evidence of invasive carcinoma in the breast and the axillary lymph nodes at the time of surgery (grade 1 and 2 of Chevalier classification).

Anthracycline containing regimens were mainly used for adjuvant and/or neo-adjuvant chemotherapy (AC60 protocol with Doxorubicin 60 mg/m2, Cyclophosphamide 600 mg/m2 and FEC 100 protocol with Fluorouracil 500 mg/m2, Epirubicin 100 mg/m2 IV, Cyclophosphamide 500 mg/m2). Docetaxel was administered at a dose of 100mg/m2 when used in monotherapy and at a dose of 75 mg/m2 in combination with Anthracycline. The choice of chemotherapy protocols depended on the availability of products at the time of the indication.

Adjuvant radiotherapy was indicated in the case of tumor size greater than 5 cm, invasion of the pectoral fascia, more than four metastatic axillary lymph nodes, positive surgical margin and breast conservation.

### Follow up

Patients were followed up until December, 2011. All patients who are not reviewed in the last consultation were contacted again by telephone. Locoregional recurrence mean the recurrence in ipsilateral mammary glands, chest wall, or regional lymph nodes identified clinically or histologically, while distant metastasis referred to the metastatic carcinoma detected by clinical examination or imaging. OS (Overall survival) was calculated from the date of diagnosis (fin needles aspiration, biopsies or radical mastectomy) to the date of death or last follow-up. DFS (Disease free survival) was measured from the date of surgery or the first course of neoadjuvant chemotherapy to the date of last follow-up or disease relapse. Patients with stage IV disease at diagnosis were excluded from the statistical evaluation of DFS. OS were calculated for all patients of the study. DFS and OS were also evaluated according to the stage for patient with localized disease.

### Statistical analysis

SPSS 18.0 software was used for statistical analysis. Descriptive of clinical data were expressed in percentage or median or mean ± SD. OS and DFS rates were estimated by Kaplan-Meier analysis.

### Consent and statement of ethical approval

As the treatment of each patient was decided by the medical staff of the centre, oral consent was obtained from the subjects and was approved by the institutional review boards of the National Institute of Oncology, Cancer Centre in Rabat. This study was approved by the institutional review boards of National Institute of Oncology, in Rabat.

## Results

### Clinical characteristics

**Figure 1:**
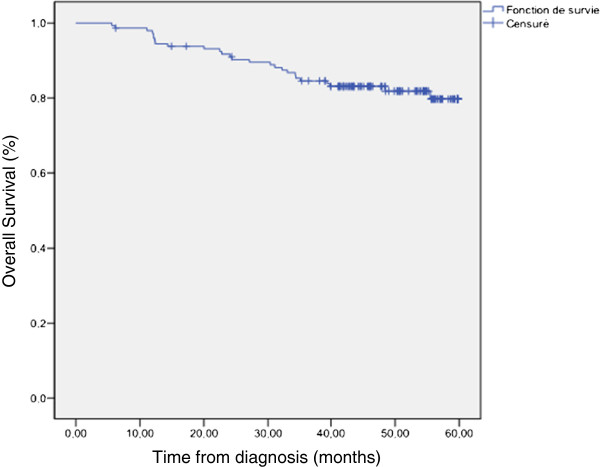
Overall survival at 5 years for all patients.

The Her2 status gene amplification was performed in Nine hundred eighty breast cancer patients treated in the national institute of oncology between January 2007 and December 2008. A total of one hundred and fifty two patients with breast cancer, were identified as having triple-negative breast cancer (16,5%). The median age at diagnosis (±standard deviation) was 46 years (range 25-79years). Twelve patients (7,8 %) had a family history of breast cancer. The identification BRCA mutation was not performed in any patient. One hundred and thirty three patients (87,5%) had nursing antecedents and thirty five patients (23%) reported the use of oral contraceptives. One hundred and thirty patients (86%) had infiltrating ductal carcinoma, thirteen had medullar carcinoma (9%), 4 had infiltrating lobular carcinoma, 3 had mixed ductal and lobular carcinomas and the two remaining patients had metaplasic carcinoma . Eighty four cases (55%) were grade III Scarff-Bloom-Richardson (SBR), 62 patients( 41%) were grade II and only six (4%) were grade I. Vascular invasion was found in 65 patients (43%). For the lymph node involvement: 48 % had positive lymph nodes, and 5 % had distant metastases. According TNM staging, 12 patients (8%) had stage I, 90 patients (60%) had stage II , 43(28%) had stage III and 7 (4%) patients had stage IV at first diagnosis. among these last patients group, three had bone metastases only, 3 had bone, lung, brain and liver metastases and one patient has diffuse cutaneous metastases. The median follow-up time was 46 months (range 6,1 -60 months). Overall survival at 5 years for all patients was 76,5% (Figure 1). Table 1 summarizes patients characteristics.

**Table 1:** Clinical characteristics and outcomes of all patients

**Characteristics**	**No. of patients**	**(%)**
Median age at diagnosis		46 years
Median histological tumor diameter		4cm
Triple negative breast cancer		16,5%
Nursing	121	59
Family history of breast carcinoma		
Yes	12	7.8
No	140	92.2
SBR grading		
I	6	4
II	62	41
III	84	55
Hsiotological type		
Invasive ductal carcinomas	130	86
Medullar carcinoma	13	9
Others types	9	5
Vascular invasion	65	43
Positive Lymphe nodes	72	49.6
M stage		
M0	145	95
M1	7	5
TNM Stage		
Stage I	12	8
Stage II	90	59
Stage III	43	28
Stage IV	7	5
Overall survival at 5 years		

### Treatment details and outcomes

#### Non metastatic disease

One hundred and forty five patients received surgery. Forty one patients (28%) had conservative surgery (tumorectomy with axillary lymph nodes). The remaining patients (72%) received radical mastectomy with axillary lymph nodes dissection (Patey type mastectomy) (Table 2). All patients with local disease, who were operated, received optimal surgery with free histological margins. Fourteen patients with advanced tumors or inflammatory breast cancer have received neoadjuvant chemotherapy before surgery (Table 2). Twelve patients (85,7%) had Anthracycline based chemotherapy (AC60and FEC100) and two patients received Anthracycline and taxane based protocol (3 cycles of AC60 folowed by 3 cycles of docetaxel). Four patients (28%) had complete response to neoadjuvant chemotherapy. Ten patients had a chevalier response 3 and 4 (37 and 63% respectively) (Table 2). From 131 patients how received adjuvant chemotherapy, 99 patients (75,5%) had Anthracycline based chemotherapy(AC60, FAC 50 and FEC100), 27 patients (20,6%) had sequential Anthracycline and docetaxel, and 4 patients (3%) received docetaxel as adjuvant chemotherapy. Seven patients received previous neoadjuvant chemotherapy (Table 2).

**Table 2:** Treatment modalities and Outcomes of non metastatic patients

	**N. of patients**	**(%)**
**Surgery**	145	
**Radical mastectomy**	104	72
**Conservative surgery**	41	28
**Chemotherapy**		
**Neoadjuvant chemotherapy**	14	10
**pCR**	4	28
**Adjuvant chemotherapy**	131	90
**Anthracycline based regimens**	99	75
**Sequentiel anthracycline taxane**	27	20
**Taxane alone**	4	5
**Outcomes**		
**Overall survival at 5 years**		79,7
**Stade I**		92,3
**Stade II**		86,5
**Stade III**		57,8
**Disease free survival at 5 years**		77,8

**Figure 2:**
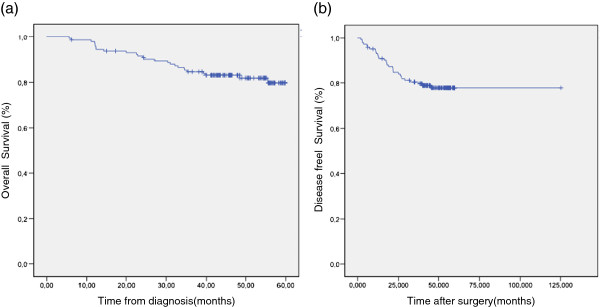
(a) overall survival at 5 years for non metastatic patients, (b) disease free survival at 5 years for non metastatic patients.

**Figure 3:**
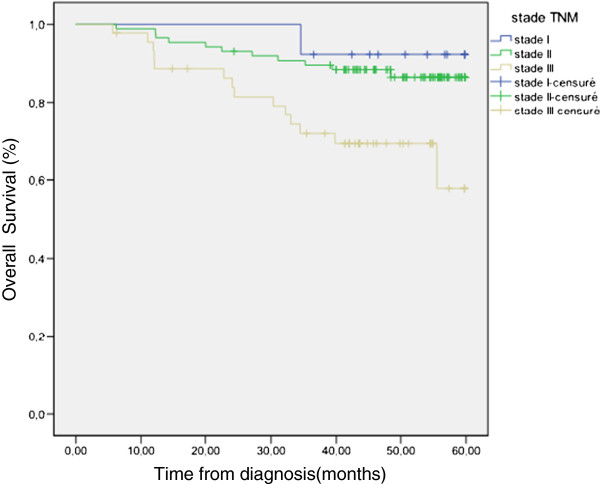
Survival for localized patients accordigng to TNM staging.

At last follow up, four patients (2,7 %) experienced local relapse, twenty seven patients (18,49%) had metastatic progression and 26 patients (17,8%) died. For all patients with localised disease, OS and DFS at 5 years were 79,7 and 77,8% respectively (Figure 2a,b) (Figure 3). OS were 92,3, 86,5 and 57,8 for stage I, II and III respectively.

#### Metastatic disease

Seven patients with metastatic disease received anthracycline-based regimen in the first line metastatic chemotherapy: four patients received AC 60 protocol, one patient received FEC100 protocol and two patients received AT protocol (Table 2). Three patients received second line chemotherapy. At the end of the study, all these patients have died.

## Discussion

This study conducted at the national institute of oncology in morocco analyzed the epidemiological, clinical, and therapeutic characteristics of TNBC. Only one recent report has evaluated the demographics and clinical presentations of this subtype in Moroccan population [13]. The current study represents a large retrospective review of 152 patients with a diagnosis of TNBC in our institution over 2-year period (2007–2008) and is to our knowledge the largest series in our context focusing on this particular population. Most of demographic and clinical features of our study group are in accordance with previous findings in the literature.

Of nine hundred eighty breast cancer patients, diagnosed between 2007 and 2008, with available immunostaining data, 16,5% were assessed as TNBC. In the US, approximately 15-20% of breast cancers are TNBC [6,7]. However, some studies have suggested that its prevalence differs between races with a higher prevalence reported among African-American women and Hispanic [8,9].

TNBC are associated with a younger age at presentation, having a mean age of 53 years old, compared to 58 years old for other subgroups in a study reported by Dent and al [7]. However, until now, there is still no final conclusion about whether age is a risk factor of TNBC, and there are still inconsistent findings in previous clinical studies. In our study population, the median age at diagnosis (46 years) was younger than the average age mostly reported in the United States [6,7] but may be comparable to the median age in Hispanic triple negative breast cancer patients [8,9]. Additionally, thirty two (17, 7%) patients had an age ≤ 35 years old suggesting that there might be factors that may predispose them to development of this disease. Given that the early age at onset is generally considered an indicator of genetic susceptibility to breast cancer and the fact that triple negative entity most often presents at a young age, an important part of future progress will be the identification of subgroups who may have a higher personal or familial risk of developing TNBC.

From an epidemiologic perspective, the known risk factors for triple-negative disease are modest, suggesting few clear interventions. Race appears to be a risk factor, as TNBC is more frequent in premenopausal patients of African-American heritage [6,10]. There is also some suggestion that TNBC are more prevalent among women who reside at a lower socioeconomic level and/or lack access to healthcare [11,12]. In the current study, no racial or socioeconomic specificity have been identified.

What is less well known is whether anthropometrics, demographics, and reproductive history are independently associated with TNBC. Previous population-based work has observed that younger age at menarche or younger age at first pregnancy, higher parity, shorter duration of breast feeding, higher body mass index (BMI) or higher waist to-hip ratio (WHR) were all associated with basal-like tumors versus luminal A tumors (tumors characterized by positive ER and PR and negative HER2) [15]. Oral contraceptive use for more than one year was associated with a 2.5-fold increase in the incidence of TNBC [16,17]. It has been proposed that the mechanism through which oral contraceptives use impacts the risk of breast cancer among young women is that estrogen promotes the growth of breast cancer-enhancing angiogenesis and stromal cell recruitment [18]. In the current series, oral contraception was found in 23% of our patients.

This study found less than 10% of family history of breast cancer. Unfortunately, the research of a BRCA1/2 gene mutation was not performed due to its non availability in the time of study period.

Recently, the association between BRCA1 mutations and the development of TNBC is well established [19]. In a recent report, Gonzalez – Angulo and al found a 19.5% incidence of BRCA mutations [20]. Review of the spectrum of breast cancer tumor subtypes, which include basal-like, triple-negative and BRCA1-positive tumors, suggest that they have overlapping clinical, pathologic and molecular features, which are different from endocrine responsive breast cancers. Gene-profiling studies of this heterogeneous subset have lead to a better understanding of the molecular pathology of these aggressive tumors and the identification of possible therapeutic targets [21]. More recently, poly (ADP-ribose) polymerase PARP inhibitors appear to take advantage of the concept of synergic lethality, or dual pathway inhibition, in attacking triple-negative and BRCA-associated tumors [22,23]. The current study found less than 10% of family history of breast cancer. Unfortunately, the research of a BRCA1/2 gene mutation was not performed due to its non availability in the time of study period.

Reviews data from others cases series show that a lower proportion of triple-negative breast cancers are discovered by mammography, which is possibly related to the age distribution of these patients [6,10]. In our study, the triple-negative breast cancers were more likely to be detected through clinical exam than through imaging, such as mammography and ultrasound. This may reflect a more rapid growth rate and the probable necessity of mammography screening in younger patient < 50 in our country.

Clinically, TNBC Patients had relatively large tumors (two thirds were >2 cm) and a high rate of node positivity (48%). Other reports have observed that patients with TNBC generally present at a similar stage compared to other tumors [7,8,16], but appear to have an inferior outcome [7,8,16,19]. This inferior prognosis has been found to be independent of several other factors such as tumor grade, size and nodal status [24].

Histologically, TNBC are associated with a higher histological grade, marked cellular pleomorphism, increase mitotic activity and atypical mitotic figures [25,26]. Similarly, in our series, TNBCs are characterized by a frequent ductal histology, high grade in two third of patient, and high proliferation and mitotic rates.

Triple negative breast cancer in the studied patients was more frequently diagnosed at advanced stage. Consequently, the majority of patients received radical mastectomy. Only 23% of our patients with local disease received conservative surgery. Haftty et all demonstrates in a previous report that there was no evidence that TNBC patients are at higher risk for local relapse after conservative surgery and radiation although triple negative have a poor prognosis [27].

Dent and al reports that the pattern of distant recurrence was strikingly different between cancer subgroups. In patients with triple negative breast cancer, the risk of any recurrence rose sharply from date of diagnosis, peaked 1 to 3 years, and dropped quickly thereafter. The patients outcomes (OS and DFS) in our series are in accordance with other previous reports [7,27].

TNBC clearly represents an important clinical challenge. This is due to poor disease-free intervals in the adjuvant and neoadjuvant setting, shortened progression-free survival associated to a more aggressive clinical course in the metastatic setting, and the lack of targeted therapy [7]. Most recent studies in triple-negative disease are directed to better identify effective treatment options and improve outcomes in these patients. Chemotherapy is known to be effective in triple-negative disease, and advances in chemotherapy have particularly benefited this patient group. However, there is not a clear, proven effective single agent that targets a driving vulnerability in triple-negative breast cancer.

Although response to chemotherapy is high in the neoadjuvant setting, the overall prognosis of this subset of tumors remains poor. Liedtke and al demonstrates in a previous report that patients with TNBC have increased pathologic complete response (pCR) rates compared with non-TNBC, and also had better survival compared to TNBC patients who don’t achieve pCR.

## Conclusion

However, a poorer prognosis was seen in the triple-negative subgroup with residual disease (RD) after completing neoadjuvant chemotherapy, particularly in the first 3 years [28]. The systemic effect of neoadjuvant chemotherapy on the immune system deserves to be accurately investigated. Several researchers have dedicated their attention to cancer immune response in order to identify prognostic factors and immunological targets [29]. In a recent study, Denkert and al reported that it is possible to identify a subgroup of breast carcinomas that is characterized by a lymphocytic infiltrate In the tumor tissue and a particularly strong response to chemotherapy [30,31].In our series, 28% of patients receiving neoadjuvant chemotherapy achieved pCR, which is in accordance with the literature [5,28].

Most approaches in triple-negative disease consist of more targeted chemotherapy, growth factor pathway approaches, and BRCA1-driven approaches. Many antiangiogenic treatments have been introduced or are currently under development, and may hold promise for patients with triple-negative breast cancer. Bevacizumab has the most data in breast cancer [30]. In a recent report from Von Minckwitz and al, the addition of bevacizumab to neoadjuvant chemotherapy significantly increased the rate of pathological complete response among patients with HER2-negative early-stage breast cancer. Efficacy was restricted primarily to patients with triple-negative tumors, in whom the pathological complete response is considered to be a reliable predictor of long-term outcome [32,33].

In metastatic setting, three randomised clinical trials (E2100, AVADO and RIBBON I) has proven the efficacy of Bevacizumab combined with chemotherapy, especially with taxanes, as the first line treatment of metastatic breast cancer. The combination improved patient progression-free survival in all trials with no impact on the known toxic effects of taxanes. This may be a potent treatment option particularly for patients with triple negative breast cancer [32,34].

As mentioned previously, PARP inhibition may be another therapeutically valuable mechanism in patients with triple negative disease. Some clinical data are available to support this approach. In one phase II study of olaparib, an oral PARP inhibitor was conducted in pretreated BRCA1 or BRCA2 mutation carriers, 41% exhibited response to this therapy [35]. Another PARP inhibitor, Iniparib, is an i.v. agent studied first in a randomized phase II of patients with metastatic triple-negative disease. Patients were randomized to receive gemcitabine plus carboplatin either alone or with the addition of Iniparib. This trial, suggested that the addition of iniparib to chemotherapy improved the clinical benefit and survival of patients with metastatic triple-negative breast cancer without significantly increased toxic effects. However, this result was not confirmed by the phase III trial presented at the 2011 ASCO Annual Meeting. [35,36]. The selection of patients who may benefit from these agents is essential, for instance, these agents may prove to be effective only in patients with BRCA1 or BRCA2 mutations

The majority of triple-negative breast cancer tumors overexpress the EGFR(epidermal growth factor receptor). EGFR has been considered as a sensible target in basal-like and triple negative breast cancer. Cetuximab, which is an anti-EGFR monoclonal antibody, was added to carboplatin therapy in a pretreated triple-negative cohort, with a modest response rate of 17% [37]. Meanwhile, in a subset of the U.S. Oncology 225200 trial, the addition of cetuximab to carboplatin and irinotecan led to a higher response rate, 49% versus 30%, but did not improve the PFS interval. However, cetuximab blocked expression of the EGFR pathway in only a minority, suggesting that most had alternate mechanisms for pathway activation [38,39].

In summary, this report documents the clinical experience of TNBC at our institution. This study is limited by our unfortunate inability to determine the exact incidence of TNBC and to do the comparative study with other breast cancer subtype. In addition, the lack of cytogenetic investigation of BRCA gene mutation due to the low socioeconomic level of these patients and the lake of Her-2 gene amplification test in the half of breast cancer patients diagnosed in our institution are two major limitations.

However, our results suggest that most TNBC characteristics in Moroccan patients are in accordance with literature data, especially concerning young age at diagnosis high grade tumors, advanced stage at diagnosis, and short time to relapse. Because mammography screening programs should improve early detection rates for breast cancer, and because the early detection of TNBC is critical for improving likelihood of its successful treatment, we believe that screening mammography is particularly important for young Moroccan women.

## Abbreviations

TNBC: Triple negative breast cancer; ER: Estrogen receptor; PR: Progesterone receptor; HER2: Human epidermal growth factor receptor 2; BMI: Body mass index; SES: Socioeconomic status; SEER: Surveillance epidemiology and end results; IHC: Immunohistochemical; EGFR: Epidermal growth factor receptor.

## Competing interests

The authors declare that they have no competing interests.

## Authors’ contributions

GR and SR were involved in the analysis of the data and the literature research, and she also wrote the manuscript. FR,SN and HA helped with the literature research. MA and SK helped with the literature research. HM and YB helped with modifications and revision of the manuscript. AJ performed the histological examination. HE approved the treatment and analyzed the literature data. All authors read and approved the final manuscript.

## Authors’ information

R.G: Resident on medical oncology at the national institute of oncology, Rabat, Morocco.

S.R: Resident on medical oncology at the national institute of oncology, Rabat, Morocco.

S.N: Medical oncologist at the national institute of oncology, Rabat, Morocco.

F.R: Resident on Radiotherapy at the national institute of oncology, Rabat, Morocco.

M.A: Resident on medical oncology at the national institute of oncology, Rabat, Morocco.

S.K: Resident on medical oncology at the national institute of oncology, Rabat, Morocco.

H.A: Medical oncologist at the national institute of oncology, Rabat, Morocco.

Y.B: Medical oncologist at the national institute of oncology, Rabat, Morocco.

B.N: Professor on pathology department at the national institute of oncology, Rabat, Morocco.

H.M: Professor on medical oncology at the national institute of oncology, Rabat, Morocco.

H. E: Professor on medical oncology at the national institute of oncology, Rabat, Morocco.

## Pre-publication history

The pre-publication history for this paper can be accessed here:

http://www.biomedcentral.com/1472-6874/12/35/prepub
